# Is “Loss of Control” Always a Consequence of Addiction?

**DOI:** 10.3389/fpsyt.2013.00036

**Published:** 2013-05-15

**Authors:** Mark D. Griffiths

**Affiliations:** ^1^International Gaming Research Unit, Psychology Division, Nottingham Trent UniversityNottingham, UK

Research into addiction has a long history although there has always been much debate as to what the key components of addiction are. Irrespective of the theory and model of addiction, most theorizing on addiction tends to assume (implicitly or explicitly) that “loss of control” is central (if not fundamental) to addiction. This short paper challenges such notions by arguing that there are a minority of individuals who appear to be addicted to a behavior (i.e., work) but do not necessarily appear to display any loss of control.

## Primary and Secondary Addictions

Research into many different types of addiction has shown that addicts are not a homogeneous group, and this may also have implications surrounding control and loss of control. Many years ago, I argued that in relation to problem gambling there appear to be at least two sub-types of addiction – primary addictions and secondary addictions (Griffiths, 1995). I defined primary addictions as those in which a person is addicted to the activity itself, and that individuals love engaging in the activity whether it is gambling, sex, or playing video games (Griffiths, 2005). Here, the behavior is primarily engaged in to get aroused, excited, and/or to get a “buzz” or “high.” I defined secondary addictions as those in which the person engages in the behavior as a way of dealing with other underlying problems (i.e., the addiction is symptomatic of other underlying problems). Here the behavior is primarily engaged in to escape, to numb, to de-stress, and/or to relax. This distinction between primary and secondary addicts shares strong conceptual, pragmatic, and theoretical similarities with other addiction typologies such as Skog’s (2003) distinction between “happy addicts” and “clinical addicts,” and the notions of positive and negative addictions as put forward by theorists such as Glasser (1976) and Rachlin (2000). In all of these typologies, whether “primary,” “happy,” or “positive,” the key characteristic is that the addict is not ambivalent about their behavior and they have not tried to change it.

Therapeutically, I argued that it is easier to treat secondary addictions (Griffiths, 1995). My argument was that if the underlying problem is addressed (e.g., depression), the addictive behavior should diminish and/or disappear. Primary addicts appear to be more resistant to treatment because they genuinely love the behavior (even though it may be causing major problems in their life). Furthermore, the very existence of primary (or positive and happy) addictions challenges the idea that loss of control is fundamental to definitions and concepts of addiction. Clearly, people with primary addictions have almost no desire to stop or cut down their behavior of choice because it is something they believe is life affirming and central to the identity of who they are. But does lack of a desire to stop the behavior they love prevent “loss of control” from occurring? Arguably it does, particularly when examining the research on workaholism (and will be returned to later in the paper).

## The Addiction Components Model

One increasingly influential model of addiction that I have popularized is the “addiction components model,” particularly in relation to behavioral addiction (i.e., non-chemical addictions that do not involve the ingestion of a psychoactive substance). The addiction components model operationally defines addictive activity as any behavior that features what I believe are the six core components of addiction (i.e., salience, mood modification, tolerance, withdrawal symptoms, conflict, and relapse) (Griffiths, 2005).

I have consistently argued that any behavior that fulfils the six criteria (outlined in more detail below) can be operationally defined as an addiction. Support for the addiction components model comes from a number of studies that have developed specific screening instruments to assess behavioral addictions, such as exercise (Terry et al., 2004; Griffiths et al., 2005), shopping (Clark and Calleja, 2008), video gaming (Lemmens et al., 2009), work (Andreassen et al., 2012a), and social networking (Andreassen et al., 2012b). My six core components of addiction (Griffiths, 2005) comprise:

- *Salience* – This occurs when the activity becomes the single most important activity in the person’s life and dominates their thinking (preoccupations and cognitive distortions), feelings (cravings), and behavior (deterioration of socialized behavior). For instance, even if the person is not actually engaged in the activity they will be constantly thinking about the next time that they will be (i.e., a total preoccupation with the activity).
- *Mood modification* – This refers to the subjective experiences that people report as a consequence of engaging in the activity and can be seen as a coping strategy (i.e., they experience an arousing “buzz” or a “high” or paradoxically a tranquilizing feel of “escape” or “numbing”).
- *Tolerance* – This is the process whereby increasing amounts of the activity are required to achieve the former mood modifying effects. This basically means that for someone engaged in the activity, they gradually build up the amount of the time they spend engaging in the activity every day.
- *Withdrawal symptoms* – These are the unpleasant feeling states and/or physical effects (e.g., the shakes, moodiness, irritability, etc.) that occur when the person is unable to engage in the activity.
- *Conflict* – This refers to the conflicts between the person and those around them (interpersonal conflict), conflicts with other activities (e.g., work, social life, hobbies, and interests) or from within the individual (e.g., intra-psychic conflict and/or subjective feelings of loss of control) that are concerned with spending too much time engaging in the activity.
- *Relapse* – This is the tendency for repeated reversions to earlier patterns of excessive engagement in the activity to recur, and for even the most extreme patterns typical of the height of excessive engagement in the activity to be quickly restored after periods of control.

One of the observations that can be made by examining these six criteria is that “loss of control” is not one of the necessary components for an individual to be defined as addicted to an activity. Although I acknowledge that “loss of control” can occur in many (if not most) addicts (Griffiths, 2005), loss of control is subsumed within the “conflict” component rather than a core component in and of itself. The main reason for this is because I believe that there are some addictions – particularly behavioral addictions such as workaholism – where the person may be addicted without necessarily losing control. However, such a claim depends on how “loss of control” is defined and the highlights the ambiguity in our standard understanding of addiction (i.e., the ambiguity of control as ability/means versus control as goal/end).

## Defining Loss of Control and the Case of Workaholism

When theorists define and conceptualize “loss of control” as applied to addictive behavior, it typically refers to (i) the loss of the ability to regulate and control the behavior, (ii) the loss of ability to choose between a range of behavioral options, and/or (iii) the lack of resistance to prevent engagement in the behavior. In some behaviors such as workaholism and anorexia, the person arguably tries to achieve control in some way (i.e., over their work in the case of a workaholic, or over food in the case of an anorexic). However, this in itself is not a counter-example to the idea that addiction is a “loss of control” if workaholics and anorexics have lost the ability to control other aspects of their day-to-day lives in their pursuit of control over work or food (i.e., there is a difference between control as the goal/end of behavior, and control as an ability/means).

There is an abundance of research indicating that one of the key indicators of workaholism (alongside such behaviors as high performance standards, long working hours, working outside of work hours, and personal identification with the job) is that of control of work activities (Porter, 1996). In a recent paper, I also noted that the need for control is high among workaholics, and as a consequence they have difficulty in disengaging from work leading to many other negative detrimental effects on their life such as relationship breakdowns (Griffiths and Karanika-Murray, 2012). Even some of the instruments developed to assess workaholism utilize questions concerning the need to be in control. For instance, Mudrack and Naughton (2001) developed a workaholism measure comprising two scales (the Non-Required Work Scale and the Control of Others Scale). The Control of Others Scale included four items reflecting the interpersonal and intrusive nature of workaholism (such as taking responsibility for the work of other people, and checking on the accuracy of other people’s work) all of which suggest a behavior that is about being in control rather than out of it. Mudrack and Naughton also reported that the Control of Others Scale correlated positively with job involvement, number of hours worked, and conflict with non-work activities. However, as noted above, the need to be in control in these examples, is not the opposite of “loss of control” as the there is a subtle difference between an individual trying to control their behavior of choice, and loss of control as relating to not being able to resist engaging in the behavior of choice.

There are also other studies that suggest some workaholics do not experience a “loss of control” in the traditional sense that is used elsewhere in the addiction literature. For instance, Mudrack (2004) reported that two particular aspects of obsessive-compulsive personality (i.e., being stubborn and highly responsible) were predictive of workaholism. Libano et al. (2010) noted that enthusiastic-type workaholics had high self-efficacy that led to high autonomy (i.e., independent, self-controlled work output). Furthermore, Tabassum and Rahman (2013) noted that perfectionist workaholics experience an overbearing need for control and are very scrupulous and detail-oriented about their work. Unusually among addictions, workaholics usually have no desire to reduce or regulate their work behavior (i.e., there is no ambivalence or conflicting desire for them). In this instance, there is no evidence of “loss of control” as traditionally understood, because if they had ambivalent or conflicting desires, they would change their behavior (i.e., reduce the amount of time they spend working). Although not an exhaustive list of studies, those mentioned here appear to indicate that some workaholics appear to be more in control than not in control.

When the addiction is primary, the goal/end of the behavior is desired and/or endorsed without ambivalence by the addict. In these situations (as in some cases of workaholism), there is no evidence for loss of control, because no (failed) attempts are made by the addict to alter their behavior. However, this could arguably still be compatible with the claim that there is loss of control in the sense of ability and/or means, because, if the workaholic tried to work less (or work in a less controlling way) because they started to recognize ill effects the addictive behavior was having on their personal life, then they may fail to do so. Therefore, the lack of evidence is indicative rather than conclusive.

However, one of the reasons that workaholism raises interesting theoretical and conceptual issues concerning the loss of control is that it is an example of an addiction where the goal/end is itself a form of control (i.e., control over their productivity/outputs, control over others, control over time-keeping, etc.). Unlike many other addictions, such behavior is not impulsive and/or chaotic but carefully planned and executed. So this raises the question, in what sense is workaholism a loss of control, understood in the typical way, as ability/means to the behavior’s goal/end? In some cases of workaholism, there is no evidence that the workaholic lacks control over this goal/end, as they do not try to change their behavior (and thus cannot fail to do so).

## Conclusion

It could be argued – and this is admittedly speculative – that “loss of control” as is traditionally understood appears to have a greater association with secondary addiction (i.e., where an individual’s addiction is symptomatic of other underlying problems) than primary (or “happy” or “positive”) addiction (i.e., where an individual feels totally rewarded by the activity despite the negative consequences). Such a speculation has good face validity but needs empirical testing. However, a complicating factor is the fact that my studies on adolescent gambling addicts have demonstrated that some individuals start out as primary addicts but became secondary addicts over time (Griffiths, 1995) – a finding that has also been applied to transitional stages of drug addictions (e.g., Koob and Le Moal, 1997). Again, this suggests that control (and loss of it) may be something that changes its nature over time.

In essence, workaholics appear to make poor choices and/or decisions that have wide-reaching detrimental consequences in their lives. However, at present we lack evidence that (should they decide otherwise) they would be unable to work in a more healthy way. Furthermore, and equally as important, the nature of workaholic behavior is not impulsive and chaotic, but carefully planned and executed. This is particularly striking among some workaholics, because as I have noted (Griffiths, 2011), it is an addiction that for some individuals they continue to work happily despite objectively negative consequences (e.g., relationship breakdowns, neglect of parental duties, etc.). What the empirical research on workaholism suggests is that it is an example of an addiction in which the problem is better characterized as loss of prudence rather than loss of control, as traditionally understood.
